# Cell-specific deletion of *C1qa* identifies microglia as the dominant source of C1q in mouse brain

**DOI:** 10.1186/s12974-017-0814-9

**Published:** 2017-03-06

**Authors:** Maria I. Fonseca, Shu-Hui Chu, Michael X. Hernandez, Melody J. Fang, Lila Modarresi, Pooja Selvan, Grant R. MacGregor, Andrea J. Tenner

**Affiliations:** 1 Department of Molecular Biology and Biochemistry, University of California, Irvine, Irvine, CA 92697 USA; 2 Department of Pathology and Laboratory Medicine, University of California, Irvine School of Medicine, Irvine, CA 92697 USA; 3 Department of Developmental and Cell Biology, University of California, Irvine, Irvine, CA 92697 USA; 4 Department of Neurobiology and Behavior, University of California, Irvine, Irvine, CA 92697 USA

**Keywords:** C1q, Alzheimer’s, Expression, Microglia, Mouse model, Complement, Conditional knockout

## Abstract

**Background:**

The complement cascade not only provides protection from infection but can also mediate destructive inflammation. Complement is also involved in elimination of neuronal synapses which is essential for proper development, but can be detrimental during aging and disease. C1q, required for several of these complement-mediated activities, is present in the neuropil, microglia, and a subset of interneurons in the brain.

**Methods:**

To identify the source(s) of C1q in the brain, the *C1qa* gene was selectively inactivated in the microglia or Thy-1^+^ neurons in both wild type mice and a mouse model of Alzheimer’s disease (AD), and C1q synthesis assessed by immunohistochemistry, QPCR, and western blot analysis.

**Results:**

While C1q expression in the brain was unaffected after inactivation of *C1qa* in Thy-1^+^ neurons, the brains of *C1qa* ^*FL/FL*^ *:Cx3cr1* ^*CreERT2*^ mice in which *C1qa* was ablated in microglia were devoid of C1q with the exception of limited C1q in subsets of interneurons. Surprisingly, this loss of C1q occurred even in the absence of tamoxifen by 1 month of age, demonstrating that Cre activity is tamoxifen-independent in microglia in *Cx3cr1* ^*CreERT2/WganJ*^ mice. C1q expression in *C1qa* ^*FL/FL*^ *: Cx3cr1* ^*CreERT2/WganJ*^ mice continued to decline and remained almost completely absent through aging and in AD model mice. No difference in C1q was detected in the liver or kidney from *C1qa* ^*FL/FL*^ *: Cx3cr1* ^*CreERT2/WganJ*^ mice relative to controls, and *C1qa* ^*FL/FL*^ *: Cx3cr1* ^*CreERT2/WganJ*^ mice had minimal, if any, reduction in plasma C1q.

**Conclusions:**

Thus, microglia, but not neurons or peripheral sources, are the dominant source of C1q in the brain. While demonstrating that the *Cx3cr1* ^*CreERT2/WganJ*^ deleter cannot be used for adult-induced deletion of genes in microglia, the model described here enables further investigation of physiological roles of C1q in the brain and identification of therapeutic targets for the selective control of complement-mediated activities contributing to neurodegenerative disorders.

**Electronic supplementary material:**

The online version of this article (doi:10.1186/s12974-017-0814-9) contains supplementary material, which is available to authorized users.

## Background

The complement system provides rapid recognition and response to danger that threatens the host. The complement cascade is regulated by the local environment [1] and synergizes with other responses to infection or stress or both [2–4]. Complement activation is carefully controlled, and imbalanced activity is associated with many degenerative diseases including rheumatoid arthritis, nephritis, traumatic brain injury, and age-related macular degeneration (reviewed in [5, 6]). The complement system also functions during development with complement-dependent synapse pruning being an integral neurodevelopmental process, at least in the brain regions such as the retinogeniculate system [7, 8]. While beneficial during development, recent reports suggest that unregulated complement-mediated synapse pruning is associated with behavioral alterations in mouse models of AD [9], aging [10], frontotemporal dementia [11], and virus infection [12]. Further delineation of the molecules in the pathway that can be most strategically targeted for therapeutic intervention in such disorders requires precise information about unintended consequences of inhibition of the entire pathways and clarification of the sources of various complement components that would enable more selective targeting to prevent or slow neurodegenerative diseases [13].

The complement system consists of over 30 independent proteins. C1q is the recognition component of C1, the classical complement pathway multi-subunit complex. C1q is a hexamer of trimers composed of three distinct polypeptide chains, A, B, and C [14], with each chain required for proper assembly of C1q [15]. When complexed with C1r and C1s, the enzymatic components of the C1 complex, C1q binding to an activator initiates a cascade of enzymatic reactions resulting in the opsonization of the activating substance, generation of the activation peptides C3a and C5a, and formation of a membranolytic pore. Binding of C3a and C5a to their cellular receptors contributes to inflammation [16]. However, C1q, independent of C1r and C1s, can enhance clearance of apoptotic cells and cellular debris, downregulate proinflammatory cytokine expression by phagocytes in vitro [17–19], and protect neurons from nutrient stress and fibrillar amyloid-induced damage [20, 21] consistent with a role in maintaining homeostasis. Indeed, synthesis of C1q is increased in response to a variety of neuronal injuries (reviewed in [5]).

To analyze the contribution of C1q to Alzheimer’s disease (AD) pathology, previous studies used a constitutive *C1qa* knockout in two mouse models of AD that demonstrated a ~50% decrease in glial activation markers and protection of neuronal integrity [22]. Subsequent work treating mouse models of AD with a C5a receptor antagonist resulted in a 50–70% decrease in pathological markers suggesting that the detrimental effects of complement activation in the brain were predominantly mediated by the proinflammatory C5a fragment generated upon the activation of the complement cascade through the cleavage of C5 (to C5a and C5b) [23]. We have previously shown that C1q synthesis is increased at an early age in AD mouse models (i.e., in the absence of fibrillar amyloid plaques), without a similar rise in C1r and C1s, which are induced only much later when the deposition of fibrillar amyloid plaques occurs [21]. Thus, with differential synthesis of complement proteins in the brain, C1q could have beneficial effects during neurodevelopment as a component of C1, be directly neuroprotective early in disease processes independent of other complement activities, and contribute to complement-mediated inflammatory events accelerating progression of cognitive loss at later stages of disease, i.e., when C1r, C1s and other complement pathway proteins are present.

To further delineate the physiological roles of C1q in the brain, including the influence of age, injury, and/or the cellular source of C1q, new models enabling defined cellular and temporal control of C1q expression are needed. Specifically, generation of mice in which C1q is present for the beneficial and protective activities during development and early stages of injury, but which could be ablated at later ages to suppress detrimental activities resulting from activation of the complement cascade would be useful. In addition, while there is evidence of *C1q* mRNA synthesis in the brain [24–26], the cellular source of C1q within the brain (neurons or microglia), and the contribution of the central nervous system (CNS) versus periphery to the production of functional C1q protein in the brain with aging and stages of disease have remained unclear. The ability to regulate detrimental responses in the brain while retaining the protective functions of the complement system in the peripheral would be beneficial particularly for long-term treatment of chronic diseases. These issues can be investigated using mice in which Cre recombinase activity can be induced in a time and cell-type specific manner. Here, we characterized C1q expression in mice homozygous for a *loxP*-flanked *C1qa* chain gene crossed to mice containing tamoxifen-inducible cre (Cre-ERT2) under control of the *Cx3cr1*, *Thy1*, *or Rosa26* promoters*.* The results, while revealing a caveat in the use of *Cx3cr1* ^*CreERT2*^, demonstrate that microglia, not neurons or peripheral sources, are the dominant source of C1q in a healthy brain at all ages up to 10 months and in an Alzheimer’s disease transgenic model.

## Methods

### Generation of mouse models

All animal experimental procedures were approved by the Institutional Animal Care and Use Committee of University of California, Irvine, and performed in accordance with the NIH Guide for the Care and Use of Laboratory Animals. The *C1qa* ^*tm1a(EUCOMM)Wtsi*^ “reporter-tagged insertional with conditional potential” allele (hereafter referred to as *C1qa* ^*GT/+*^) is a gene-trap allele in which a Frt’d splice-acceptor–*lacZ*–polyadenylylation (polyA) cassette and PGK-neo/G418 resistance cassette is inserted within intron 2 of *C1qa* (see https://www.mousephenotype.org/data/genes/MGI:88223) (obtained from Jackson Laboratory, Bar Harbor, ME). This allele is predicted to be null for *C1qa* function. To analyze *lacZ* (β-galactosidase; β-gal) reporter gene expression from the targeted allele of *C1qa*, mice with a *C1qa* ^*GT/+*^ allele were crossed with B6.FVB-Tg(*Stra8-cre*)/1Reb/LguJ mice (Jackson, stock #017490). This removes the PGK-neo cassette that is flanked by *loxP* sites, to avoid possible interference of the PGK-neo cassette with transcription of the *C1qa*-encoded *lacZ* reporter gene. Offspring with the *C1qa* ^*tm1b(EUCOMM)Wtsi*^ “reporter-tagged deletion” allele, hereafter referred to as *C1qa* ^*GT-neo/+*^, retain one wild type *C1qa* allele to prevent developmental abnormalities that may result from a complete deletion of C1q during development. To generate mice with a *cre-*conditional (“floxed”) allele, *C1qa* ^*GT/+*^ were crossed with B6.Cg-Tg(*ACTFLPe*)9205Dym/J (Jackson, stock # 005703) mice to produce mice with a *C1qa* ^*tm1c(EUCOMM)Wtsi*^ allele, hereafter referred to as *C1qa* ^*FL/+*^. In the *C1qa* ^*FL/+*^ allele, the coding sequence in exon 3 is flanked by two *loxP* sites, one within intron 2 and the other in the 3′ untranslated region of exon 3. To generate mice for tissue- and temporal-specific knockout of *C1qa*, *C1qa* ^*FL/FL*^ mice were crossed with B6.129P2(Cg)-*Cx3cr1* ^*tm2.1(cre/ERT2)Litt*^/WganJ, (Jackson, stock #021160) mice, here designated as *Cx3cr1* ^*CreERT2/WganJ*^ or *Cx3cr1* ^*CreERT2*^, which co-expresses Cre-ER fusion protein and EYFP in monocytes, dendritic cells, NK cells, and brain microglia [27], or Tg(*Thy1-cre/ERT2,-EYFP*)HGfng/PyngJ, (Jackson, stock #012708), here referred to as *Thy1* ^*CreERT2*^ which expresses Cre-ERT2 fusion protein and EYFP in Thy1-expressing neurons, or B6.129-*Gt(ROSA)26Sor* ^*tm1(cre/ERT2)Tyj*^/J, (Jackson, stock #008463), here designated as *Rosa26* ^*CreERT2*^ mice which expresses Cre-ERT2 fusion protein ubiquitously. All mice were on the C57BL6/J background (i.e., *Nnt* ^*−/−*^). Progeny from each cross were backcrossed with *C1qa* ^*FL/FL*^ mice to generate *C1qa* ^*FL/FL*^ *:Cx3cr1* ^*CreERT2*^, *C1qa* ^*FL/FL*^ *:Thy1* ^*CreERT2*^, and *C1qa* ^*FL/FL*^ *:Rosa26* ^*CreERT2*^ animals. In addition, *Cx3cr1* ^*CreERT2*^ were crossed to B6;129S6-Gt(ROSA)26Sortm14(*CAG-tdTomato*)Hze/J (Jackson, stock #007908, also called Ai14(RCL-tdT)-D), which we refer to as *ROSA26-STOP-tdTomato*, to generate *ROSA26-STOP-tdTomato +/−: Cx3cr1* ^*CreERT2*^ mice. As a sensitive cell lineage reporter of Cre activity, cells with a *ROSA26-STOP-tdTomato* allele do not express tdTomato until after the floxed stop cassette has been excised by Cre recombinase activity. Arctic48 mice were obtained from Dr. Lennart Mucke (Gladstone Institute, San Francisco, CA, USA). Constitutive *C1qa* knockout mice [15] were originally a gift from Dr. Marina Botto, Imperial College London.

### Induction of Cre activity with tamoxifen

Tamoxifen (T5648; Sigma, St. Louis, MO) was dissolved in 5% ethanol/corn oil at final concentration of 50 mg/ml. Mice were treated with tamoxifen at 0.2 mg/g body weight or vehicle control by oral gavage once a day for five consecutive days [28]. Animals were bled, and tissue was harvested at 3–56 days after treatment as noted.

### Collection of tissue

Tissue was processed as previously described [23]. Briefly, mice were anesthetized with isoflurane, perfused with PBS, and the brains were collected and fixed for 24 h in 4% paraformaldehyde/PBS. To detect C1q in interneurons, mice were perfused with PBS, followed by 4% paraformaldehyde/PBS for 2 min. Tissue was then post-fixed for only 2 h in 4% paraformaldehyde/PBS at 4 °C. For some mice, the liver and kidney were also collected.

### Immunohistochemistry

Immunostaining procedures were performed as described [22]. Briefly, 40-μm brain or 30-μm liver and kidney were incubated overnight at 4 °C with primary antibodies or corresponding control IgG. Primary antibodies were detected with Alexa555 or Alexa488 labeled secondary antibodies (Invitrogen, Carlsbad, CA). Antibodies used were anti-mouse C1q (rabbit monoclonal, clone 27.1) tissue culture supernatant (previously generated and characterized in collaboration with the Barres lab [29], anti-Cre recombinase (mouse monoclonal ascites, clone 2D8, 1:1000, Millipore, Temecula, CA), anti GAD67 clone (mouse monoclonal clone 1G10.2, 1:700, Millipore ), anti-parvalbumin (mouse monoclonal, clone PARV-19, 1:300, Sigma), anti-somatostatin (rat monoclonal, clone YC7, 1:200, Millipore), anti-F4/80 (rat monoclonal clone CI:A3-1, 0.5 ug/ml, AbD Serotec), anti-CD31 (rat monoclonal, clone SZ31, 1 ug/ml, Dianova, GmbH, Germany), and anti-Iba-1 (rabbit polyclonal, 1 ug/ml, Wako, Richmond, VA). For colocalization experiments of C1q with other markers, primary antibodies were incubated simultaneously (with anti-Cre recombinase, F4/80, CD31, somatostatin, or parvalbumin) or sequentially (after anti-GAD67) followed by secondary antibodies. For immunoperoxidase staining of C1q, CD45 or amyloid ß, sections were pretreated with 3%H_2_O_2_/10%MeOH/Tris-buffered saline (TBS), pH 7.4 to block endoperoxidase. After blocking with 2%BSA/10% normal goat serum/0.1%Triton/TBS, sections were incubated with anti-mouse CD45 (goat polyclonal 1 ug/ml, R&D) or anti-amyloid ß (#1536, 1:1,000, gift of Dr. Cooper [30]) in blocking solution or anti-mouse C1q clone 27.1, overnight at 4 °C. After incubation with primary antibody, sections were incubated with the corresponding biotinylated secondary antibodies (Vector Labs, Burlingame, CA) (1 h, RT) followed by ABC (Vector Labs) (1 h, RT) and developed with DAB (3,3′-diaminobenzidine) (Vector Labs) following manufacturer instructions. Tissue was dehydrated and mounted with DePeX (BDH Laboratory Supplies, Poole, England). Immunostaining was analyzed using a Zeiss Axiovert-200 inverted microscope (Zeiss, Thornwood, NY) and images acquired with a Zeiss Axiocam high-resolution digital camera (1300x1030 pixel) using Axiovision 4.6 software. Confocal images were acquired using a Zeiss LSM 700 or 780 confocal microscopes and Zen software. Images of staining were acquired with the same exposure times and camera settings when comparing the same brain area in the different genotypes. The percentage of C1q-positive microglia (yellow) over total microglia (green + yellow) per animal was obtained by scoring the number of yellow and green microglia per field and averaging all five fields per section (two sections per animal). For quantitative analysis of C1q in the molecular layer of hippocampus, images were analyzed using ImageJ software. Four regions of interest (ROI) (squares) were defined randomly within the molecular layer area and the mean pixel intensity per ROI was determined. The mean intensity of each animal was obtained by averaging all ROI mean intensities in the sections studied (two–three sections per animal). For CD45 and Aß, images were analyzed using Axiovision 4.6 sofware. Percentage of immunopositive area (immunopositive area/total image area times 100) was determined by averaging several images per section (*n* = 2–3 sections per animal) that covered the hippocampal area. One way ANOVA statistical analysis was used to assess the differences in C1q mean intensity, plaque area, and glial reactivity.

### ß-gal staining

Detection of *lacZ* (ß-gal) expression by X-Gal histochemistry was done using a staining kit (Life Technologies, Carlsbad, CA). Sections were incubated with a final concentration of 1 mg/ml X-gal, 4 mM potassium ferricyanide, 4 mM potassium ferrocyanide, and 2 mM magnesium chloride, with or without 25 μM sodium deoxycholate at RT or 37 °C for different times (between 30 min and 24 h). Detection of ß-gal by immunostaining was performed using a rabbit polyclonal anti-ß-galactosidase (0.1–10 ug/ml) (Life Technologies) or chicken polyclonal anti-ß galactosidase (1:100–1:5000, Abcam, Cambridge, MA).

### Microglial isolation, sorting, and immunocytochemistry

Primary neonatal microglia were isolated as described [31] from postnatal day 2 pups from a *C1qa* ^*FL/FL*^ *:Cx3cr1* ^*CreERT2*^ animal crossed with *C1qa* ^*FL/FL*^. Cells were stained with an Allophycocyanin (APC)-conjugated anti-mouse CD11b. Viable cells were gated according to size and granularity (based on forward and side scatter properties), followed by gates for YFP and CD11b (FACS Aria II). The sorted populations, (1) YFP^+^, CD11b^+^; (2) YFP^−^, CD11b^+^; and (3) YFP^−^, CD11b^−^, were plated on poly-l-lysine coated coverslips for 1 h. After blocking with 2% BSA/0.1% Triton/PBS for 30 min and incubating with or without rabbit monoclonal anti-C1q antibody (clone 27.1) for 1 h at RT, followed by Alexa 555 goat anti-rabbit antibody (1 h, RT), coverslips were mounted with antifade reagent containing DAPI (Prolong gold, Invitrogen). No APC or Alexa 555 signal was detected when the primary anti-C1q antibody was omitted.

### Hemolytic titer

Plasma was collected into EDTA (10 mM final) immediately preceding perfusions by cardiac puncture. The blood was withdrawn from the left ventricle with a 25-gauge needle and transferred to a prechilled glass tube on ice. After centrifugation at 2000×*g* for 10 min at 4 °C, the plasma was removed and immediately distributed into small aliquots and stored at −80 °C. Two microliters of mouse plasma diluted in GVB (4.8 mM sodium barbital, 0.1% gelatin, 142 mM NaCl, 1 mM MgCl_2_, and 0.15 mM CaCl_2_ at pH 7.3) was combined with human C1q-deficient serum (300 μl). Eighty microliters of sheep erythrocytes (Colorado Serum Co., Denver, CO) previously sensitized with rabbit anti-sheep erythrocyte immunoglobulin (EA) (5 × 10^8^ EA/ml) was added and incubated at 37 °C for 30 min. After addition of 1.6 ml ice-cold GVB, reactions were centrifuged at 800×*g* at 4 °C for 3 min and the optical density at 412 nm of the supernatants were obtained as an index of released hemoglobin. The functional activity of mouse C1q was determined by analysis of hemolytic activity derived from the means of duplicate serial dilutions of each sample as previously described [32].

### Western blot

Each frozen half-brain or hippocampus was pulverized, aliquoted, and stored at −80 °C. Brain powder was solubilized by homogenization in ten volumes of Tris-buffered saline (TBS, pH 7.4) containing protease inhibitor cocktail solution (Complete mini, Roche), 1 mM EDTA, and 2% SDS using motor pestle for 5 s twice on ice. After centrifugation at 18,400×*g* for 30 min at 4 °C, protein concentration in the supernatants was determined with BCA protein assay (Pierce, Rockford, IL). Sixty μg of brain protein or 1 ul plasma were subjected to SDS-polyacrylamide gel electrophoresis (10%) under reducing conditions. Gels were transferred at 4 °C in Tris-glycine, SDS, and 10% methanol transfer buffer onto polyvinylidenedifluoride (PVDF, Immobilon-P, Millipore) membrane at 300 mA for 2 h. PVDF membrane was blocked 1 h at RT in 5% nonfat dry milk in TBS-Tween. Rabbit anti-mouse C1q (1151) [33] or mouse anti-ß-actin (Sigma) in either 5 or 3% milk respectively was applied overnight at 4 °C or 2 h at RT. After washing, HRP-conjugated secondary antibodies diluted 1:5000 (Jackson Labs), were added and incubated for 1 h at RT. The blots were developed using ECL 2 or ECL (Pierce) and analyzed using a Nikon D700 digital camera and the ImageJ software as described [34].

### RNA extraction and qRT-PCR

Total RNA from pulverized mouse brain (5–10 mg) was extracted using the Illustra RNAspin Mini Isolation Kit (GE Healthcare). cDNA synthesis was performed using SuperScript III reverse transcriptase (Life Technologies) according to the manufacturer’s protocol. Quantitative RT-PCR was performed using the iCycler iQ and the iQ5 software (Bio-Rad) with the maxima SYBR/Green Master Mix (Thermo Fisher Scientific). The mouse primers for *C1qa* [21], *C1qb*, and *C1qc* genes [35] and *Hprt* primers were obtained from Eurofins MWG Operon (Huntsville, AL). *Hprt* primers were designed using primer-blast (http://www.ncbi.nlm.nih.gov/tools/primer-blast/), with the forward sequence (5′-3′): AGCCTAAGATGAGCGCAAGT and reverse sequence (5′-3′): ATCAAAAGTCTGGGGACGCA. The relative mRNA levels were determined as follows: mRNA levels = 2^-ΔCt^ and ΔCt = (Ct_Target_−Ct_HPRT_). Ct values represent the number of cycles at which fluorescence signals were detected [36].

## Results

*C1q in the brain is colocalized with microglia*, *neuropil*, *and interneurons but not Thy1+ neurons.*

Initial attempts to use the *lacZ* reporter detection in the C1q “reporter-first” gene-targeted mouse failed to provide a signal in the brain (Additional file 1), and thus, the monoclonal anti-C1q antibody clone 27.1 was used to analyze C1q expression. As reported [29], this antibody stained microglia, interneurons, and was particularly strong in the molecular layer of hippocampus (Additional file 1: Figure S1A–C). As a negative control for antibody specificity, tissue from a homozygous constitutive *C1qa* knockout animal was routinely stained in parallel and showed complete lack of reactivity with this antibody (Additional file 1: Figure S1D).

**Fig. 1:**
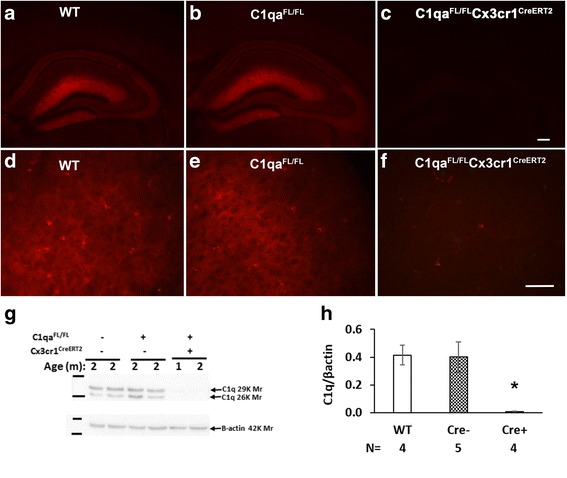
Expression of C1q is present at normal levels in the brains of *C1qa* ^*FL/FL*^ mice but nearly absent in *C1qa* ^*FL/FL*^ *:Cx3cr1* ^*CreERT2*^ mice independent of tamoxifen administration. C1q staining in hippocampus of **a** WT, **b** *C1qa* ^*FL/FL*^, and **c** *C1qa* ^*FL/FL*^ *:Cx3cr1* ^*CreERT2*^ mice and in the cortex of **d** WT, **e** *C1qa* ^*FL/FL*^, and **f** *C1qa* ^*FL/*FL^ *:Cx3cr1* ^*CreERT2*^ in the absence of tamoxifen treatment. Representative images of *n* = 3–4 animals per genotype (ages 3–6 m) in (**a**–**f**). Scale bars: **a**–**c**:200 um, **d**–**f**: 50 um. **g** Representative western blots of total brain protein extract (60 ug per lane) from WT, *C1qa* ^*FL/FL*^, and *C1qa* ^*FL/FL*^:*Cx3cr1* ^*CreERT2*^, run under reducing conditions and probed with polyclonal anti-mouse C1q (1151) and anti-actin, as a loading control. **h** Densitometric ratio of C1q/ß-actin from several western blots performed as in (**g**), presented as the means +/− SEM of 4–5 animals per group as noted. *P < .03 relative to either WT or C1q^FL/FL^ lacking Cre by one-way ANOVA followed by Bonferroni’s multiple comparisons test

Before investigating the source of synthesis of C1q protein in the brain, we first verified that expression of C1q in mice homozygous for a floxed allele of *C1qa* (hereafter referred to as *C1qa* ^*FL/FL*^) was unchanged compared to WT using immunofluorescence (Fig. 1a, b, d, and e) and western blot analysis of whole brain extracts (Fig. 1g, h). We also verified that *Cx3cr1* ^*CreERT2WganJ*^ mice [27] (hereafter called *Cx3cr1* ^*CreERT2*^) and *Thy1* ^*CreERT2*^ [37] mice used to induce cell-specific ablation of the *C1qa* gene expressed the CreERT2 fusion protein (as well as EYFP) as expected in microglia and neurons respectively in the brain. First, YFP, co-expressed with CreERT2 under transcriptional control of the *Cx3cr1* locus in *Cx3cr1* ^*CreERT2*^ mice, colocalized exclusively with all Iba1-positive cells (microglia) (Additional file 1: Figure S2A) as predicted since in the brain, Cx3CR1 is expressed in all microglia [38], and thus, YFP can be considered a marker for microglia in this model. In addition, Cre colocalized with YFP^+^ cells (microglia) in *Cx3cr1* ^*CreERT2*^ mice (Additional file 1: Figure S2B), as expected from the construct. Similarly, since Thy1 is a neuron-specific promoter (present in projection neurons) Cre and YFP co-label neurons in *Thy1* ^*CreERT2*^ mice as shown in the cortex (Additional file 1: Figure S2C).

**Fig. 2:**
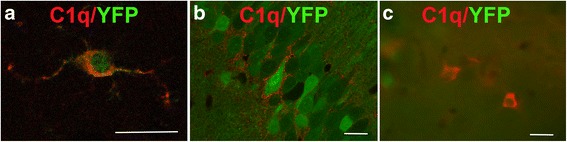
C1q colocalizes with YFP in microglia of *Cx3cr1* ^*CreERT2*^ mice but not with YFP in *Thy1* ^*CreERT2*^ mice. Colocalization of C1q (*red*) with **a** YFP (*green*) in *Cx3cr1* ^*CreERT2*^ mice or **b**, **c** YFP (*green*) in the *C1qa* ^*FL*^ *:Thy1* ^*CreERT2*^ mice. **a** *Cx3cr1* ^*CreERT2*^ 8 m, **b** *C1qa* ^*FL/+*^ *:Thy1* ^*CreERT2*^ CA1, 4 m, **c** *C1qa* ^*FL/FL*^ *:Thy1* ^*CreERT2*^ hippocampus, 9 m. Representative pictures of *n* = 3–4 animals per genotype. Scale bar (**a**–**c**): 20 um

We also confirmed that C1q colocalizes with YPF (Fig. 2a) inside cell bodies of *Cx3cr1* ^*CreERT2*^ microglia, consistent with previous demonstration of C1q colocalization with CX3CR1^GFP^-positive microglia [29]. In *Thy1* ^*CreERT*^ , mice where YFP is expressed in Thy1^+^ neurons, C1q did not colocalize within YFP^+^ cells but instead was detected in the neuropil surrounding YFP-labeled neurons (Fig. 2b) and in the synaptic layers of the hippocampus as previously seen in wild type mice [29]. The extracellular location of the C1q in these regions was confirmed by analysis of confocal Z-stack images of C1q staining in *Thy1* ^*CreERT2*^ mice (Additional file 1: Figure S3A). In the *Thy1* ^*CreERT2*^ mice, in addition to microglia, C1q staining was present in cells that were YFP negative and that morphologically resembled interneurons (Fig. 2c) and colocalized with GAD67, an intracellular interneuron-specific marker (Additional file 1: Figure S3B). Some, but not all, of the C1q-positive interneurons colocalized with parvalbumin or somatostatin (Additional file 1: Figure S4).

*C1q is unchanged in the brains of C1q* ^*FL/FL*^ *:Thy1* ^*CreERT2*^ *mice treated with tamoxifen, but is significantly reduced in the brain of C1q* ^*FL/FL*^ *:Cx3cr1* ^*CreERT2*^ *mice even without tamoxifen treatment.*

**Fig. 3:**
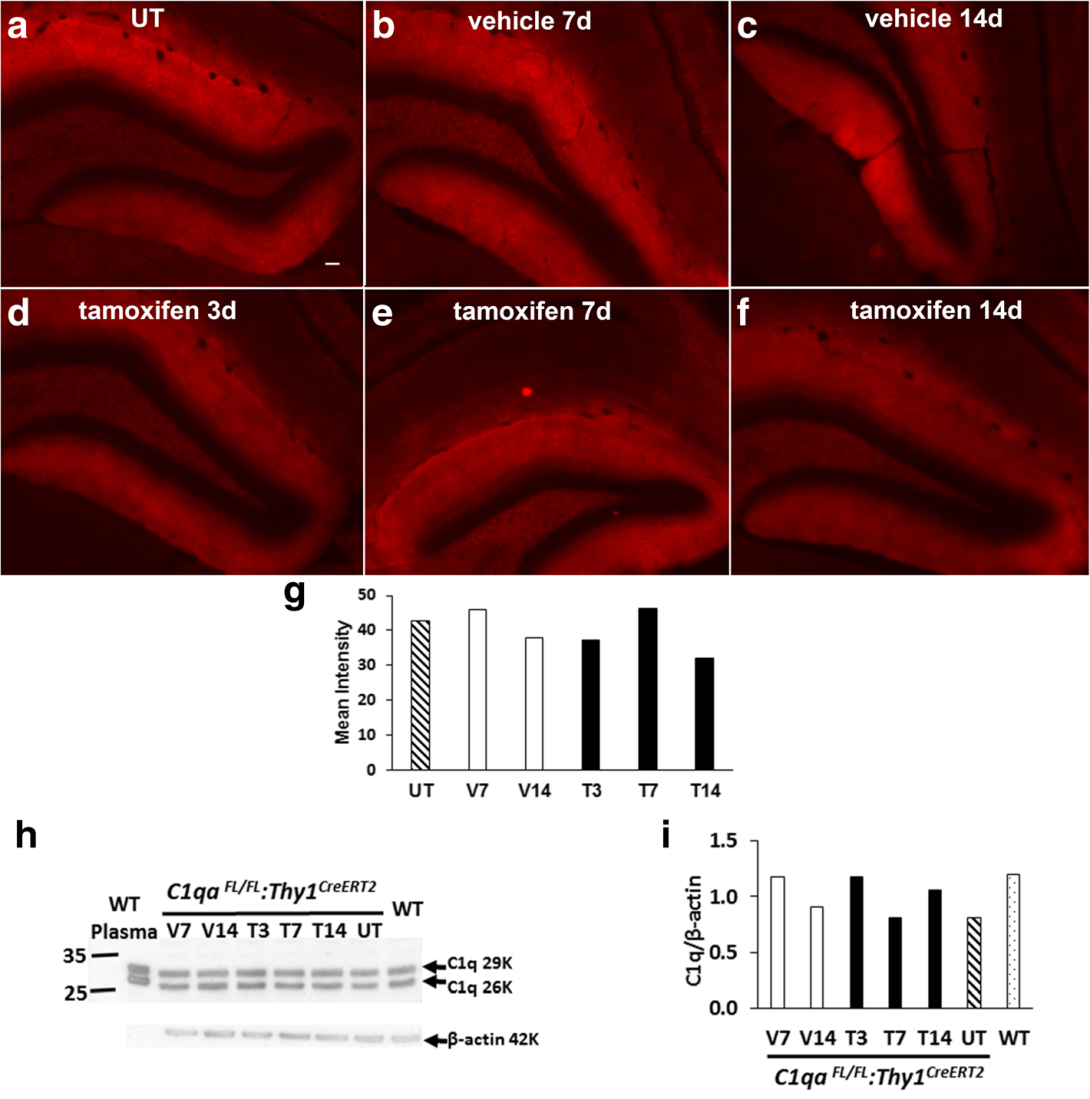
Tamoxifen treatment of *C1qa* ^*FL/FL*^ *:Thy1* ^*CreERT2*^ mice did not change levels of C1q in the brain. C1q reactivity (*red*) in hippocampus of untreated (UT) WT (**a**) and *C1qa* ^*FL/FL*^ *:Thy1* ^*CreERT2*^ (**b**–**f**) mice treated with vehicle (**b**, **c**) or tamoxifen (**d**, **e**, **f**) for 5 days and perfused at 3,7,or 14 days after treatment. Age 5 m. Scale bar: 50 μm in (**a**); same magnification in (**a**–**f**). Mean intensity of C1q immunofluorescence (**g**) in the molecular layer of hippocampus of pictures shown in panels in (**a**–**f**). Brain extracts (60 ug per lane) (**h)** from *C1qa* ^*FL/FL*^:*Thy1* ^*CreERT2*^ treated with vehicle (V) or tamoxifen (T) as in (**a**–**f**), C1q WT at 5 m, and wild type (WT) plasma (1 ul), were run under reducing conditions and probed with polyclonal anti-C1q (1151) and anti actin, as a loading control. Densitometric ratio (**i)** of C1q/ß-actin of blot in (**h**)

In animals with the *Cx3cr1* ^*CreERT2*^ or *Thy1* ^*CreERT2*^, the fusion protein CreERT2 is Cre fused to a mutant estrogen receptor (Cre^ERT2^) that binds tamoxifen but not endogenous estrogen [39]. As a result, the Cre^ERT2^ is predicted to remain in the cytoplasm until exposure to tamoxifen, at which time the Cre^ERT2^ moves into the nucleus enabling the recombination between *loxP* sites, eliminating the intervening DNA sequence. To enable temporal and cell-specific deletion of the *C1qa* gene, Cre deleters were crossed to *C1qa* ^*FL/FL*^ to generate *C1qa* ^*FL/FL*^ *:Cx3cr1* ^*CreERT2*^ and *C1qa* ^*FL/FL*^ control littermates (without *Cx3cr1* ^*CreERT2*^ *)* or *C1qa* ^*FL/FL*^ *:Thy1* ^*CreERT2*^ and *C1qa* ^*FL/FL*^ littermates without *Thy1* ^*CreERT2*^. Mice were treated with tamoxifen to delete *C1qa* in microglia or Thy1+ neurons (i.e., *C1qa* ^*FL/FL*^ *:Cx3cr1* ^*CreERT2*^ and *C1qa* ^*FL/FL*^ *:Thy1* ^*CreERT2*^, respectively). Animals (4–7 m old) were analyzed between 3–14 days after treatment. Neither *C1qa* ^*FL/FL*^ *:Thy1* ^*CreERT2*^ nor control *C1qa* ^*FL/FL*^ mice showed a decrease in C1q in the brain after tamoxifen administration compared with vehicle controls up to 14 days post treatment by both immunofluorescence analyses (Fig. 3a–g) and western blot analysis of whole brain extracts (Fig. 3h, i).

While analyzing the impact of deleting the *C1qa* gene in microglia on total brain C1q protein, C1q staining in *C1qa* ^*FL/FL*^ *:Cx3cr1* ^*CreERT2*^ adult animals was seen to be very low or absent, even at the earliest timepoint after tamoxifen treatment (*n* = 15, data not shown). Surprisingly, vehicle treated (*n* = 9, data not shown) and the untreated *C1qa* ^*FL/FL*^ *:Cx3cr1* ^*CreERT2*^ animals (Fig. 1c, f) also lacked C1q reactivity throughout the brain as shown in both the molecular layer of the hippocampus (Fig. 1c) and in the cortex (Fig. 1f) in contrast to WT (Fig. 1a, d) or *C1qa* ^*FL/FL*^ littermate mice (Fig. 1b, e). This dramatic decline was confirmed by western blot of brain extracts in 1-and 2-month-old animals (Fig. 1g, h). C1q was detected in a subset of interneurons (GAD67+) in the *C1qa* ^*FL/FL*^ *:Cx3cr1* ^*CreERT2*^ mice, although the intensity was decreased in comparison to interneurons in the littermates lacking *Cx3cr1* ^*CreERT2*^ (Additional file 1: Figure S4A, right panel).

**Fig. 4:**
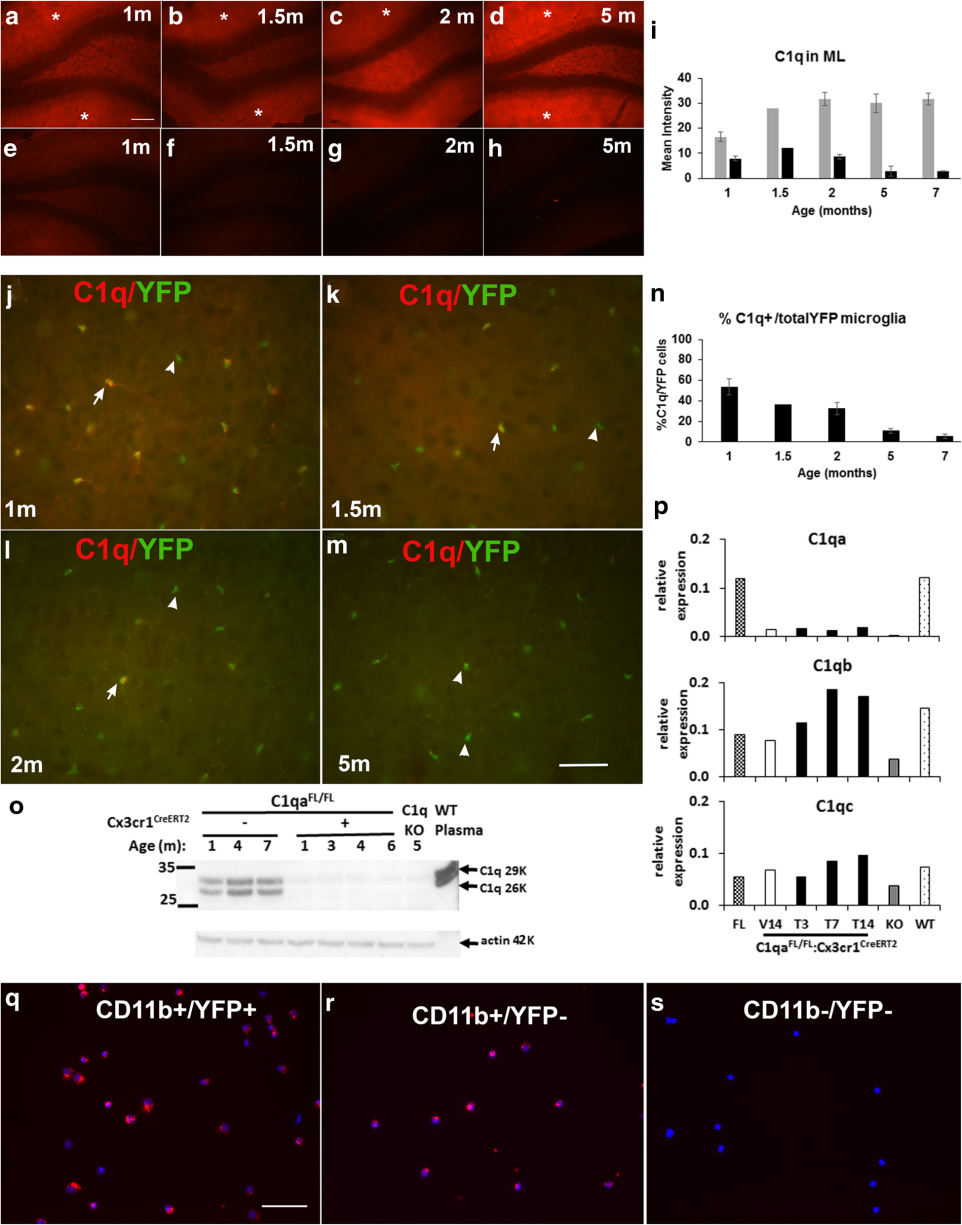
C1q reactivity is present in neonatal microglia of *C1qa* ^*FL/FL*^ *:Cx3cr1* ^*CreERT2*^ pups, but is decreased at 1 month and continues to decline with age in *C1qa* ^*FL/FL*^ *:Cx3cr1* ^*CreERT2*^. Representative images of C1q immunostaining (*red*) in the hippocampus (**a**–**h**) and cortex (**j**–**m**) of *C1q* ^*FL/FL*^ (**a**, **b**), WT (**c**, **d**), or *C1q* ^*FL/FL*^ *:Cx3cr1* ^*CreERT2*^ (**e**–**h**, **j**–**m**) at different ages as noted in the panels. Identical exposure time and illumination settings were used for each image in (**a**–**h**) and similarly were identical for the images in (**j**–**m**). Merged images of C1q immunostaining (*red*) and YFP (*green*) showing C1q colocalization with microglia (*yellow*, *arrows*) or progressive lack of C1q (*YFP green* only, *arrowheads*) in *C1q* ^*FL/FL*^ *Cx3cr1* ^*CreERT2*^ mice with age (**j**–**m**). Average of the mean intensity of C1q immunostaining in the molecular layer (*) of *C1qa* ^*FL/FL*^ *(grasy)* or *C1qa* ^*FL/FL*^:*Cx3cr1* ^*CreERT2*^ (*black*) at different ages (1 month, *n* = 4; 1.5 months, *n* = 1; 2 months, *n* = 2; 5 months, *n* = 3; 7 months, *n* = 2), respectively (**i**). *p* values obtained from data from 3–4 mice at 1 and 5 months by ANOVA single factor analysis are *p* = 0.02 and 0.005, respectively. Quantification of the percentage of total microglia that were C1q positive in the cortex of *C1qa* ^*FL/FL*^:*Cx3cr1* ^*CreERT2*^ mice (**n**). Percentage of C1q-positive microglia per animal was obtained by averaging five images per section per mouse. *Bar* represent average % of C1q-positive microglia in n mice per age (1 month, *n* = 4; 1.5 months, *n* = 1; 2 months, *n* = 2; 5 months, n = 3; 7 months, *n* = 2). Brain extracts (60 ug per lane) from *C1qa* ^*FL/FL*^ and *C1qa* ^*FL/FL*^:*Cx3cr1* ^*CreERT2*^ littermates at 1 to 7 months of age, C1qKO at 5 m, and wild type (WT) plasma (1 ul) (**o**), were run as in Fig. 1. Representative of 3–9 animals per genotype. *C1qa*, *C1qb*, and *C1qc* mRNA relative to *Hprt* (**p**) in the brain from *C1qa* ^*FL/*FL^ (FL), *C1qa* ^*FL/FL*^:*Cx3cr1* ^*CreERT2*^ at 3, 7, or 14 days after treatment with vehicle (V) or tamoxifen (T), *C1qa*KO (KO), and wild type (WT) mice by qPCR. Cells isolated from the brains of 2-day-old *C1qa* ^*FL/FL*^ *:Cx3cr1* ^*CreERT2*^ (YFP+) and *C1qa* ^*FL/FL*^ (YFP−) mice were stained with anti-CD11b, sorted by CD11b and YFP and subsequently stained with anti-C1q (*red*) and DAPI (*blue*) (**q**–**s**). Representative pictures of microglia (CD11b+) that are (**q**) *Cx3cr1* ^*CreERT2*^ (YFP+) show similar C1q staining compared to (**r**) *C1qa* ^*FL/*FL^ (YFP−) microglia (CD11b+). No C1q is present in populations negative for CD11b and YFP (neurons, astrocytes) (**s**). Scale bar 100 μm (**a**–**h**) or 50 μm (**j**–**m**,**q**–**s**)

To determine if the tamoxifen-independent deletion of C1q in microglia and neuropil was age dependent, the brain of animals of 1, 1.5, 2, 5, and up to 7 months were examined for C1q reactivity. By 1 month of age, there was a profound decrease of C1q in the molecular layer of hippocampus (Fig. 4e vs a) of *C1qa* ^*FL/FL*^ *:Cx3cr1* ^*CreERT2*^ mice, that further decreased with age (Fig. 4f–i) compared with WT (Fig. 4c, d) or littermate *C1qa* ^*FL/FL*^ lacking *Cx3cr1* ^*CreERT2*^ (Fig. 4a, b, i). Similarly, C1q-positive microglia (which remain YFP positive due to the YFP expressed under *Cx3cr1* ^*CreERT2*^ *)* decreased dramatically by 1–1.5 months of age as seen in the cortex (Fig. 4j–k) and was essentially negative at all ages thereafter (Fig. 4l–m) as quantified in Fig. 4n. However, Iba-1-positive microglia (that are also YFP+, Additional file 1: Figure S2A) were not reduced in *C1qa* ^*FL/FL*^ *:Cx3cr1* ^*CreERT2*^ (data not shown). The absence of C1q seen by immunofluorescence in the brain of *C1q* ^*FL/FL*^ *:Cx3cr1* ^*CreERT2*^ mice was confirmed by lack of detection of C1q protein by western blot (WB) in brain extracts (Fig. 4o). Finally, qRT-PCR to detect mRNA for the *C1qa* chain was dramatically reduced in the brain of both vehicle and tamoxifen treated *C1qa* ^*FL/FL*^ *:Cx3cr1* ^*CreERT2*^, though not to zero as found in the brains from constitutive homozygous *C1qa* knockout mice (Fig. 4p, top panel). Messenger RNA for the *C1q*b and *C1qc* chains was detected in all animals, including the constitutive *C1qa* KO (Fig. 4p), demonstrating the specificity of the recombination events.

As complement-mediated synapse pruning has been demonstrated as a normal process in neurodevelopment, it is important to demonstrate if the *C1qa* gene is deleted or still present in neonatal microglia in *C1qa* ^*FL/FL*^ *:Cx3cr1* ^*CreERT2*^ mice to perform this beneficial function. Cells from the brains of postnatal day 2 litters containing both *C1qa* ^*FL/FL*^ *:Cx3cr1* ^*CreERT2*^ (YFP+) and *C1qa* ^*FL/FL*^ (YFP-) pups were sorted for CD11b (i.e., microglia) and YFP (indicating the presence of Cre). C1q staining was clearly detected in both CD11b^+^ populations (Fig. 4q, r) with an equal percentage of CD11b^+^ cells (microglia) expressing C1q (96%) in YFP+ (*C1qa* ^*FL/FL*^ *:Cx3cr1* ^*CreERT2*^) and YFP− (*C1qa* ^*FL/FL*^ *)* microglia demonstrating that Cre deletion of the *C1qa* gene had not yet occurred in microglia from neonatal mice. All CD11b-negative cells lacked C1q reactivity as expected (Fig. 4s).

*Upregulated C1q expression in the brain from aged mice and an Alzheimer’s disease mouse model is also abrogated in C1q* ^*FL/FL*^ *:Cx3cr1* ^*CreERT2*^ *mice independent of tamoxifen treatment.*

**Fig. 5:**
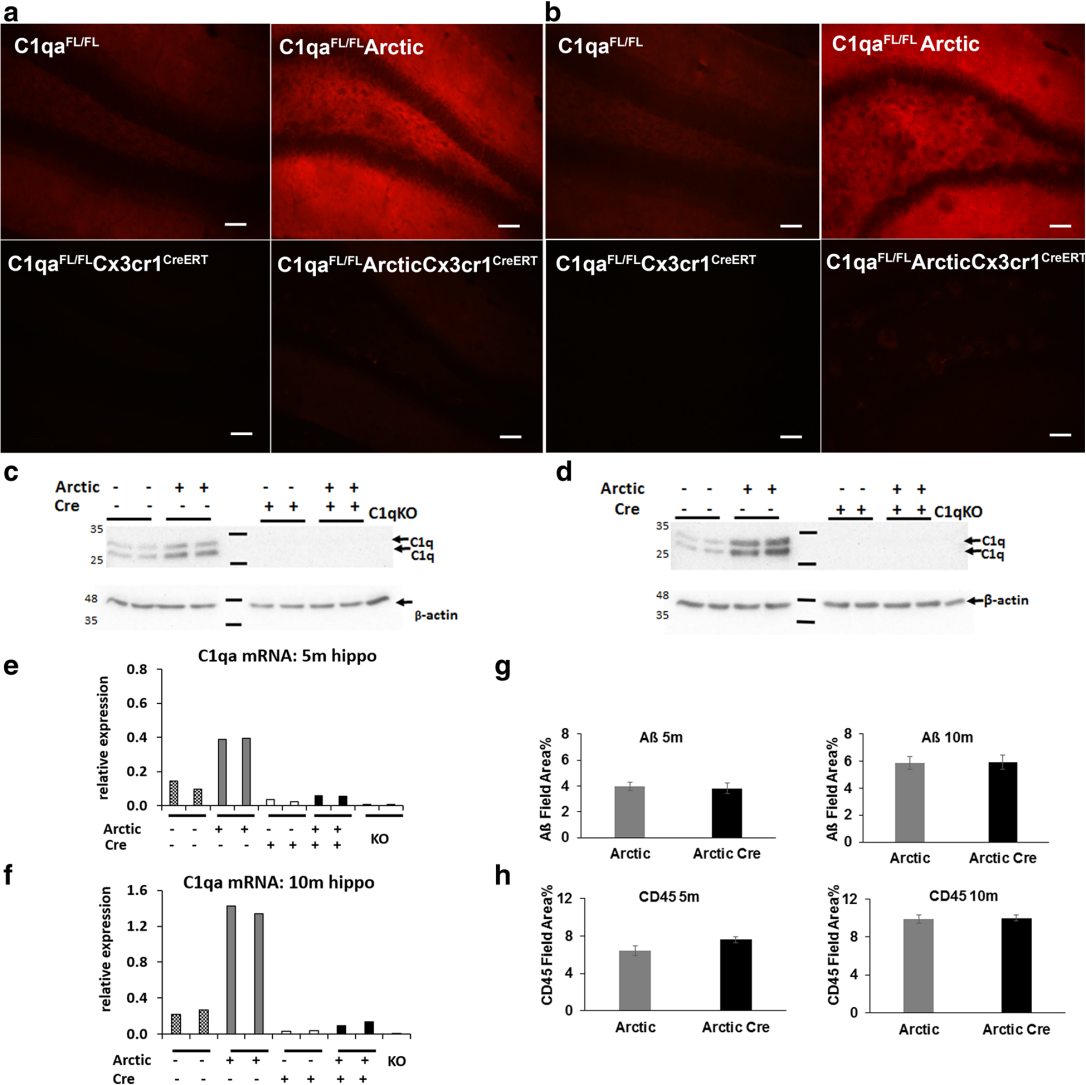
C1q reactivity in the hippocampus is nearly absent in AD *C1qa* ^*FL/FL*^ mouse models crossed to *C1qa* ^*FL/FL*^:*Cx3cr1* ^*CreERT2*^ mice. **a**, **b** C1q immunostaining (*red*) in the brains of *C1qa* ^*FL/FL*^ (*left column*) and Arctic *C1qa* ^*FL/FL*^ (*right column*) without (*top row*) or with *Cx3cr1* ^*CreERT2*^ (*bottom row*) at 5 (**a**) and 10 m (**b**) of age. Representative images of the molecular layer of 8–10 animals per genotype per age. Acquisition time and camera gain are identical for each panel within (**a** or **b**). The exposure time was shorter in (**b**) than in (**a**) to avoid overexposure due to higher levels of C1q in the Arctic brain at 10 months than at 5 months of age (**c** and **d**). Scale bars: 50 um. Western blot analysis for C1q in hippocampal extracts (60 ug per lane) from *C1qa* ^*FL/FL*^, *Arctic:C1qa* ^*FL/FL*^, *C1qa* ^*FL/FL*^:*Cx3cr1* ^*CreERT2*^ and *Arctic*:*C1qa* ^*FL/FL*^:*Cx3cr1* ^*CreERT2*^ at **c** 5 and **d** 10 months of age. Representative of 4 animals per genotype per age. **e**, **f** Increased *C1qa* mRNA expression relative to *Hprt* in hippocampal extracts from Arctic compared *C1qa* ^*FL/FL*^, mice at both **e** 5 months and **f** 10 months, with almost complete absence in all animals containing *C1qa* ^*FL/FL*^:*Cx3cr1* ^*CreERT2*^. Representative of 2–4 animals per genotype for 5mo and 3 animals per genotype for 10mo old mice. **g** Quantification (% Field Area) of amyloid and **h** CD45 staining in Arctic *C1qa* ^*FL/FL*^ with and without Cx3cr1^CreERT2^ at 5 (*left*) and 10 (*right*) months of age. *n* = 8–10 mice per genotype per age. Not statistically different by genotype at each age as assessed by one-way ANOVA (CD45 *p* = 0.10 (5 months) and *p* = 0.9 (10 months); Aß *p* = 0.8 (5 months) and *p* = 0.95 (10 months)

Previous studies have demonstrated that C1q expression increases with age and with diseases such as Alzheimer’s disease in both human and mouse models [26, 29, 40]. To determine whether increased synthesis of C1q occurs within microglia or other cell types, mice hemizygous for an *App* ^*Arctic48*^ transgene (*Arctic+*) that produces a mouse model of Alzheimer’s disease [41, 42] were used to generate *Arctic+*: *C1qa* ^*FL/FL*^:*Cx3cr1* ^*CreERT2*^ and *Arctic+*: *C1qa* ^*FL/FL*^, *C1qa* ^*FL/FL*^:*Cx3cr1* ^*CreERT2*^ and *C1qa* ^*FL/FL*^ (control) littermates that were aged to 5 and 10 m. Increased expression of C1q was clearly evident with age and presence of Arctic transgene in both the hippocampus (Fig. 5a–f) and cortex (data not shown). In contrast, little to no C1q was detected in the brains from both Arctic *C1qa* ^*FL/FL*^:*Cx3cr1* ^*CreERT2*^ and *C1qa* ^*FL/FL*^:*Cx3cr1* ^*CreERT2*^ littermates as assessed by immunofluorescence (Fig. 5a, b), western blot analysis (Fig. 5c, d), and qPCR for *C1qa* mRNA expression (Fig. 5e, f). The absence of C1q in the Arctic:*C1qa* ^*FL/FL*^:*Cx3cr1* ^*CreERT2*^ model indicates that the increased C1q expression in Arctic AD mouse model requires expression of C1q from *Cx3cr1 +* cells (i.e., microglia). Total amyloid beta and CD45 levels in the Arctic mice were not statistically different from Arctic:*C1qa* ^*FL/FL*^:*Cx3cr1* ^*CreERT2*^ at either 5 or 10 months of age (Fig. 5g, h), suggesting that in this model the near complete lack of C1q in the brain does not affect plaque pathology.

*C1q in the liver*, *kidney*, *and plasma is unaffected in C1qa* ^*FL/FL*^ *: Cx3cr1* ^*CreERT2*^ *mice.*

**Fig. 6:**
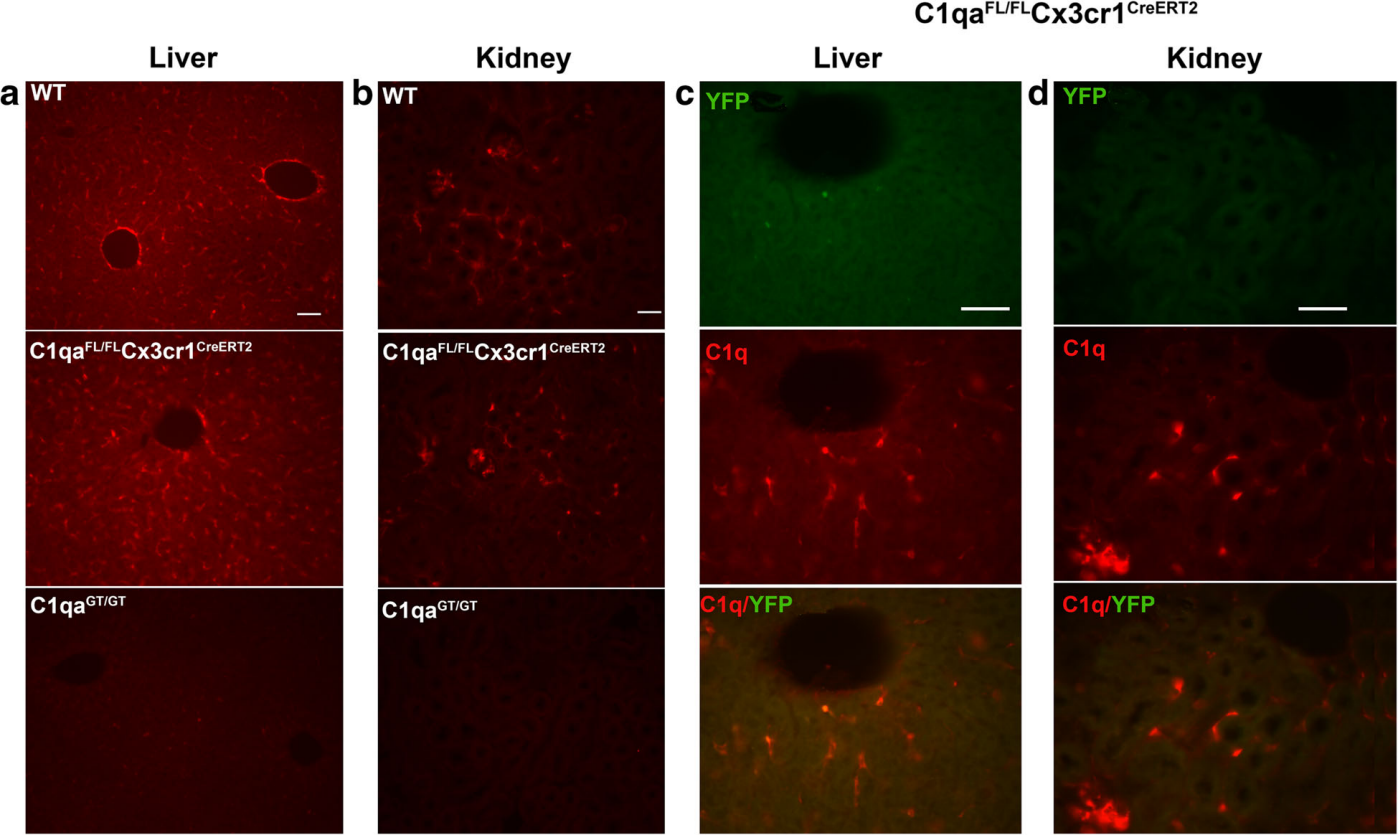
No overt difference in expression of C1q in liver cells and kidney macrophages in *C1qa* ^*FL/*FL^:*Cx3cr1* ^*CreERT2*^ compared to wild type mice, correlating with lack of YFP/Cre expression in these tissues in *C1qa* ^*FL/FL*^:*Cx3cr1* ^*CreERT2*^. C1q staining of the liver (**a**) or kidney (**b**) WT (*top panels*), *C1qa* ^*FL/FL*^ *:Cx3cr1* ^*CreERT2*^ (*middle panels*). C1q reactivity is absent from C1q^GT/GT^ (*bottom panels*) as expected demonstrating specificity of the antibody in these tissues. Representative pictures of *n* = 2–5 mice per genotype per age at 4–6 months. C1q (*red*) but not YFP (*green*) is detected in the liver (**c**) and kidney (**d**) of *Cx3cr1* ^*CreERT2*^ mice (4 months). *Bottom panels* of (**c** and **d**) are the merged images of C1q and YFP. Scale bars: 50 um

As some peripheral myeloid populations are known to express *Cx3cr1*, the potential contribution of C1q produced by the liver and kidney or other sources to brain C1q was investigated. C1q staining in the liver (Fig. 6a) and kidney (Fig. 6b) from *C1qa* ^*FL/FL*^ *: Cx3cr1* ^*CreERT2*^ and control animals at multiple ages from 1 to 10 months of age (when brain C1q was barely detectable) showed no difference in the level of C1q staining relative to WT (Fig. 6a, b; compare middle with top panels) or to *C1qa* ^*FL/FL*^ littermates lacking the *Cx3cr1* ^*CreERT2*^ allele (data not shown). No staining was present in the original gene trapped (C1qa^GT/GT^) mice as expected (Fig. 6a, b, bottom panels), validating the anti C1q antibody specificity in these tissues. Both the liver and kidney from *C1qa* ^*FL/FL*^ *:Cx3cr1* ^*CreERT2*^ mice showed that C1q colocalizes with F4/80 (Additional file 1: Figure S5A, B, respectively), although not all C1q-positive cells were F4/80 positive, consistent with the presence of subsets of myeloid cells in the liver [43]. No colocalization of C1q with the endothelial cell marker CD31 was seen (Additional file 1: Figure S5C) and confirmed by confocal analysis (data not shown). While some expression of the highly photostable GFP protein under transcriptional control of the *Cx3cr1* locus is detected in these tissues [44] (and data not shown), relatively little if any YFP is detected in the liver and kidney (Fig. 6c, d vs brain microglia YFP in Fig. 4), consistent with low to no *Cx3cr1*-driven Cre^ERT2^ [45], the retention of *C1qa* gene and thus expression of C1q in these cells.

**Fig. 7:**
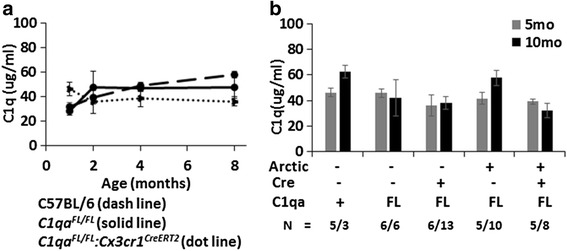
C1q concentration is similar in the blood of *C1qa* ^*FL/FL*^:*Cx3cr1* ^*CreERT2*^ and *C1qa* ^*FL/FL*^ *littermates lacking Cx3cr1* ^*CreERT2*^. **a** Compiled densitometry of western blot analysis of plasma (1 ul loaded per lane under reducing conditions) from a total of 82 animals: *C1qa* ^*FL/FL*^:*Cx3cr1* ^*CreERT*2^ (*dotted line*, *N* = 5, 5, 8, and 19), *C1qa* ^*FL/FL*^ littermates (*solid line*, *N* = 3, 4, 8, and 10), and *C57BL6/J* (*dashed line*, *N* = 2, 5, 10, and 3). Plotted values for 4 and 8 months are pooled data from 3–5 and 7–10 months of age, respectively. Membranes were probed with rabbit anti-mouse C1q antibody (1151). Values are +/− SEM. Concentration was determined relative to known wild type plasma C1q concentration on each gel. No significant difference was observed between the groups by one-way ANOVA followed by Bonferroni’s multiple comparisons test (GraphPad). **b** The same analysis was performed for 5 months (*gray*) and 10 months (*black*) *C57BL6/J*, *C1qa* ^*FL/FL*^, Arctic *C1qa* ^*FL/FL*^, *C1qa* ^*FL/FL*^:*Cx3cr1* ^*CreERT*2^, and *Arctic:C1qa* ^*FL/FL*^:*Cx3cr1* ^*CreERT*2^, all in the absence of tamoxifen treatment. There are no statistical differences in plasma C1q concentration among these ages and genotypes, using one-way ANOVA with Bonferroni’s multiple comparisons test

To determine if the level of C1q in plasma was affected in *C1qa* ^*FL/FL*^ *:Cx3cr1* ^*CreERT2*^ mice, both western blot analysis of C1q in plasma and hemolytic functional activity were assessed. C1q hemolytic activity in the *C1qa* ^*FL/FL*^ *:Cx3cr1* ^*CreERT2*^ mice (untreated, tamoxifen, or vehicle treated *n* = 20), which were negative for C1q in the brain, had only partial, if any, reduction in C1q plasma levels (data not shown). Plasma C1q protein concentration as measured by western blot analysis in *C1qa* ^*FL/FL*^ (*n* = 25) and *C1qa* ^*FL/FL*^ *:Cx3cr1* ^*CreERT2*^ (*n* = 37) mice were comparable in range to WT (*n* = 20) (Fig. 7a). Western blot analysis of plasma concentrations of C1q in aged and Arctic (AD model) mice at 5 and 10 months also demonstrated no change in C1q levels in the presence of the *C1qa* ^*FL/FL*^ *:Cx3cr1* ^*CreERT2*^ (Fig. 7b). All *C1q* ^*Fl/FL*^ mice crossed to *Thy1* ^*CreERT2*^ (with or without tamoxifen treatment) mice reflected the same range of plasma C1q concentrations as littermate controls lacking the Cre construct (data not shown). In all cases tested, relative levels of hemolytic activity matched the protein levels determined by western blot. These data demonstrate that the C1q protein in the uninjured brain, aged brain, or brain stressed by the presence of amyloid plaque and microglial activation is not derived from the blood, but rather from CX3CR1 expressing cells within the CNS itself (i.e., microglia).

*Cre activity from the Cx3cr1* ^*CreERT2*^ *allele is “leaky” in the absence of tamoxifen in long-lived microglia.*

Similar to the *Thy1* ^*CreERT2*^ mice, *C1qa* ^*FL/FL*^ mice crossed to *Rosa* ^*CreERT2*^ (ubiquitously expressed Cre-ERT2) had no difference in the level of C1q in the brain in the absence of tamoxifen at any ages tested (2–6 m). Importantly, C1q was reduced in the brain after tamoxifen treatment of *C1qa* ^*FL/FL*^: *Rosa* ^*CreERT2/+*^ mice (Additional file 1: Figure S6). These results suggest the robust Cre^ERT2^ expression driven by the *Cx3cr1* promoter compared to the relatively weak promoter of the *Rosa26* locus that is responsible for the tamoxifen-independent gene deletion, and not an inherent property of *C1qa* ^*FL/FL*^. To further test this prediction, we looked for evidence of *Cx3cr1* ^*CreERT2*^-mediated *loxP* recombination using mice containing a different floxed sequence. The *ROSA26-STOP-tdTomato* allele has a “floxed” stop cassette upstream of a coding sequence for tdTomato, such that cells express tdTomato only after the stop cassette has been removed by Cre activity. Tamoxifen-independent tdTomato fluorescence was observed in microglia in *ROSA26-STOP-tdTomato* crossed to *Cx3cr1* ^*CreERT2*^ mice by 1 and 2 months of age, consistent with leakage of the recombinase activity to the nucleus in these cells (Additional file 1: Figure S7). The percentage of YFP-positive cells that were also tdTomato-positive cells was 60.15% +/−SE 2.6 (*n* = 2) at 1 month and 73.6%, +/−SE 0.9 (*n* = 2) at 2 months, compared to 0% of *Rosa26-STOP-tdTomato mice* littermate controls lacking the *Cx3cr1* ^*CreERT2*^. Together, these results demonstrate that the tamoxifen-independent recombination of *loxP* sites mediated by the *Cx3cr1* ^*CreERT2*^ allele is not specific to the *C1qa* gene, and that this *Cx3cr1* ^*CreERT2*^, but not all Cre^ERT2^ deleter mice show C1q gene ablation in microglia in the absence of the inducer tamoxifen. That is, *Cx3cr1* ^*CreERT2WgaJn*^ is “leaky”, with Cre activity accessing the nucleus in the absence of tamoxifen, which compromises its use in studies involving postnatal temporal induction of Cre deletion of floxed genes.

## Discussion

Cell-specific Cre-recombinase-mediated ablation of the *C1qa* gene was used to demonstrate that microglia, but not Thy1+ neurons or peripheral blood, are the dominant source of C1q in mouse brain beyond one month of age. The data also indicate that the enhanced expression of C1q with aging and as seen in AD models is also predominantly, if not exclusively, microglial. Upon deletion of microglial *C1qa*, C1q is only detected at very low levels in subpopulations of interneurons in the brain. Given the known functions of C1q in clearing apoptotic cells while suppressing proinflammatory cytokine production (reviewed in [46]) and signaling direct neuroprotection [21], a dominant microglial synthesis of C1q is consistent with a reparative and protective role for microglia and the importance of an anti-inflammatory environment to maintain normal brain function. Yet, when infection or damage increases such that other complement proteins are produced, as occurs in neurodegenerative diseases, more powerful effector mechanisms can be enabled, including activation of the complement cascade with potential collateral progressive inflammatory polarization of microglia with damaging consequences.

The site of synthesis of complement proteins was traditionally believed to be in the myeloid compartment of the liver [47]. Petry and colleagues demonstrated that wild type bone marrow transplanted into a constitutive C1q knockout mouse could completely restore the plasma compartment of C1q [48]. However, in the past two decades there have been multiple reports of increases in C1q synthesis upon injury, particularly in the nervous system (reviewed in [49]) but also in other tissues [50, 51]. The upregulation of mRNA for complement components in the brain with age and in neurodegenerative diseases such as AD has been documented in both human [26, 52] and mouse models [53, 54] of disease. Evidence for both microglial and neuronal synthesis have been presented [7, 24, 55, 56]. Here, when *C1qa* ^*FL/FL*^ mice were crossed with animals expressing Cre^ERT2^ under control of the *Cx3cr1* locus (*Cx3cr1* ^*CreERT2*^), within 1 month of age, most of the brain C1q was eliminated, while no alteration in C1q protein was detected in the brain after tamoxifen treatment of mice containing a transgene expressing a tamoxifen-inducible *Cre* ^*ERT*2^ under the neuronal *Thy1* promoter.

Surprisingly, the deletion of the *C1qa* gene in *C1qa* ^*FL/FL*^ *: Cx3cr1* ^*CreERT2*^ mice occurred even in the absence of the tamoxifen suggesting that the Cre^ERT2^ gains access to the nucleus independently of tamoxifen to cause the deletion. Similar tamoxifen-independent Cre-recombinase activity was demonstrated with a different floxed sequence in microglia from *ROSA26-STOP-tdTomato:Cx3cr1* ^*CreERT2*^ (Additional file 1: Figure S7). Thus, these data suggest that the *Cx3cr1* ^*CreERT2WganJ*^ mouse does not maintain normal expression of floxed genes in microglia in the absence of tamoxifen, thereby limiting its use for inducible deletion of microglial genes. The original report describing this *Cx3cr1*-driven Cre deleter investigated only young *Cx3cr1* ^*CreERT2*^ mice treated with tamoxifen [27].

Importantly, in peripheral tissue macrophages and Kupffer cells with a shorter lifespan and lower *Cx3cr1* expression, reduction of C1q expression was not detected. In addition, microglia in *C1qa* ^*FL/FL*^ mice that also had the ubiquitously expressed *Rosa26* ^*CreERT2*^ allele also did not show a decrease in C1q in the absence of tamoxifen suggesting that in contrast to *Cx3cr1* ^*CreERT2*^, the level of expressed Cre^ERT2^ under control of the *Rosa26* promoter does not result in substantial entry of the recombinase into the nucleus to result in a detectable reduction of C1q. We hypothesize that the greater longevity of microglia combined with the prominent expression of CX3CR1 in microglia enables sufficient Cre^ERT2^ to enter the nucleus of these cells, deleting the floxed *C1qa* gene, thereby abrogating synthesis of C1q in those cells permanently. As the animals age, without turnover/renewal of this long-lived microglial population, cells with the deleted floxed gene will accumulate abrogating specific protein expression. Similar “leakiness” of a different inducible Cre^ERT^ under the robust GFAP promoter has been reported in Bergmann glial cells [57]. Thus, while microglia-specific gene ablation was achieved, temporal regulation of C1q synthesis could not be controlled by proposed inducible *Cx3cr1* ^*CreERT2*^ due to tamoxifen-independent Cre-dependent ablation in these cells within a few weeks of birth. Table 1 summarizes the effects of different inducible Cre^ERT2^ tested here.

**Table 1:** Effect of Promoter Specific Cre^ERT2^ Expression

Model	Target Gene	Cre construct	Tamoxifen^a^	Effect on target gene expression^b^					
				IHC		Western blot		QPCR	Titer
				Brain	Liver Kidney	Brain	Blood	Brain	Blood
C57Bl6J	C1qa	*Thy1* ^*CreERT2*^	-	-		-	-		-
			+	-		-	-		-
C57Bl6J	C1qa	*Cx3cr1* ^*CreERT2/WganJ*^	-	↓	-	↓	-	↓	-
			+	↓	-	↓	-	↓	-
C57Bl6J	C1qa	*Rosa26* ^*CreERT2*^	-	-			-		
			+	↓			↓		
Arctic	C1qa	*Cx3cr1* ^*CreERT2/WganJ*^	-	↓		↓	-	↓	
C57Bl6J	*CAG-tdTomato*	*Cx3cr1* ^*CreERT2/WganJ*^	-	↑^c^					

^a^"-" indicates no treatment, "+" indicates Tamoxifen treatment as described in Materials and Methods

^b^"-" indicates no change from wild type control and arrow down indicates decreased C1q

^c^evidence of nuclear recombinase activity

Interestingly, recent studies have shown that activation of the C1 complex results in the covalent tagging of synapses with the activation fragments C3b and iC3b, marking them for elimination by microglia via the complement receptor CR3 [8]. While initially discovered as critical for proper brain development (reviewed in [58]), overzealous or inappropriate “pruning” of synapses has now been associated with loss of function or alterations in behavior in mouse models of frontotemporal dementia [11], epilepsy [59], AD [9], and West Nile Virus infection [12]. However, it is also well supported that the activation of the complement cascade can lead to the generation of C5a which can recruit glia, promote their proinflammatory polarization (reviewed in [49]), and could also be a cause of the loss of phenotypic functions [23] noted in these systems. Future studies will be important to determine the relative contribution of the increased synapse elimination versus complement activation leading to C5a-enhanced inflammation/injury to these behavioral impairments. In addition, since evidence of localized neuronal C1q has been reported, it remains to be tested if specific deletion of microglial C1q will abrogate complement-dependent synapse pruning or if local synthesis of C1q may be induced in neurons during development, infection or other disease states which we did not assess here, with physiological consequences.

## Conclusions

The data presented here show that microglia, not peripheral blood or other CNS cells, are the overwhelming source of C1q in the murine brain from postnatal through the aging adult (at least though 10 months of age) and in one mouse model of Alzheimer’s disease, where C1q levels are normally dramatically increased with age and amyloid accumulation. Although these data demonstrate that loss of C1q in microglia occurs after 1 month of age with the *Cx3cr1* ^*CreERT*2/*WganJ*^ strain [27] independent of tamoxifen and thus temporal induction is not possible, this cell-specific deleter strain of mice may be valuable to determine if the cellular source of C1q (and indeed any microglia-expressed gene) in vivo changes in various models of neurological disorders and will enable subsequent investigation of the beneficial or detrimental contribution of CNS C1q to the progression of neurodegenerative diseases, including cognitive decline. We also show that it is possible to deplete the brain of C1q selectively without depleting C1q in the blood, thus enabling the delineation of C1q-mediated events as being of CNS rather than peripheral origin. These results provide a characterized framework for further investigation of beneficial roles as well as detrimental effects of complement in the CNS (reviewed in [60]), clarification of the contribution of peripheral complement to neurodegenerative diseases [13, 61], determination of what controls the induction of C1q synthesis in the brain, and subsequent design of selective and specific therapeutic interventions for complement-mediated events in neurodegenerative diseases [62].

## Additional file

Additional file 1: Failure to detect *lacZ* activity in the C1q “reporter-first” gene-targeted mouse, *C1qa* ^*tm1a(EUCOMM)Wtsi*^. (DOCX 3 mb)

## Abbreviations

AD: Alzheimer’s disease
CNS: Central nervous system
TBS: Tris-buffered saline
