# Supplementary material for: Cell-specific deletion of C1qa identifies microglia as the dominant source of C1q in mouse brain
Source: J Neuroinflammation. 2017 Mar 6;14:48. doi: 10.1186/s12974-017-0814-9 (PMC5340039; doi:10.1186/s12974-017-0814-9)
Supplement: Additional file 1: — Failure to detect lacZ activity in the C1q “reporter-first” gene-targeted mouse, C1qa tm1a(EUCOMM)Wtsi. (DOCX 3 mb) [file 12974_2017_814_MOESM1_ESM.docx]

**Additional file 1:**

Cell specific deletion of *C1qa* identifies microglia as the dominant source of C1q in mouse brain.

Maria I. Fonseca1, Shu-Hui Chu^1^, Michael X. Hernandez^2^, Melody J. Fang^1^, Lila Modarresi^1^, Pooja Selvan^1^, Grant R. MacGregor^3^ and Andrea J. Tenner^1,2,4,*^

**Failure to detection *lacZ* activity in the C1q "reporter-first" gene targeted mouse, *C1qa^tm1a(EUCOMM)Wtsi^***

To study the source of C1q in the brain, heterozygous reporter mice (*C1qa^GT/+^* ) were obtained and *C1qa^GT-neo/+^* were generated in which the *lacZ* gene was under the control of the *C1q* promoter. Brains were stained with the β-gal substrate (X-gal) or with anti-β-galactosidase, but neither detection system provided a signal above background seen in wild type tissue (i.e. lacking the *lacZ* gene-trap construct) either in brain or liver (data not shown). As a positive control, X-gal staining was seen in embryonic brains of mice expressing BRE-gal (BMP signaling reporter mice ^1^) stained in parallel. Thus, the lack of X-gal staining in these mice with the *C1q* reporter allele indicates that the reporter expression is too weak to serve as a read out of *C1qa* transcriptional activity either due to inefficient translation initiation at the *lacZ* coding sequence in intron 2, or relatively low transcriptional activity from the *C1qa* promoter or that intronic sequences needed for sufficient *lacZ* expression were disrupted by the inserted construct.


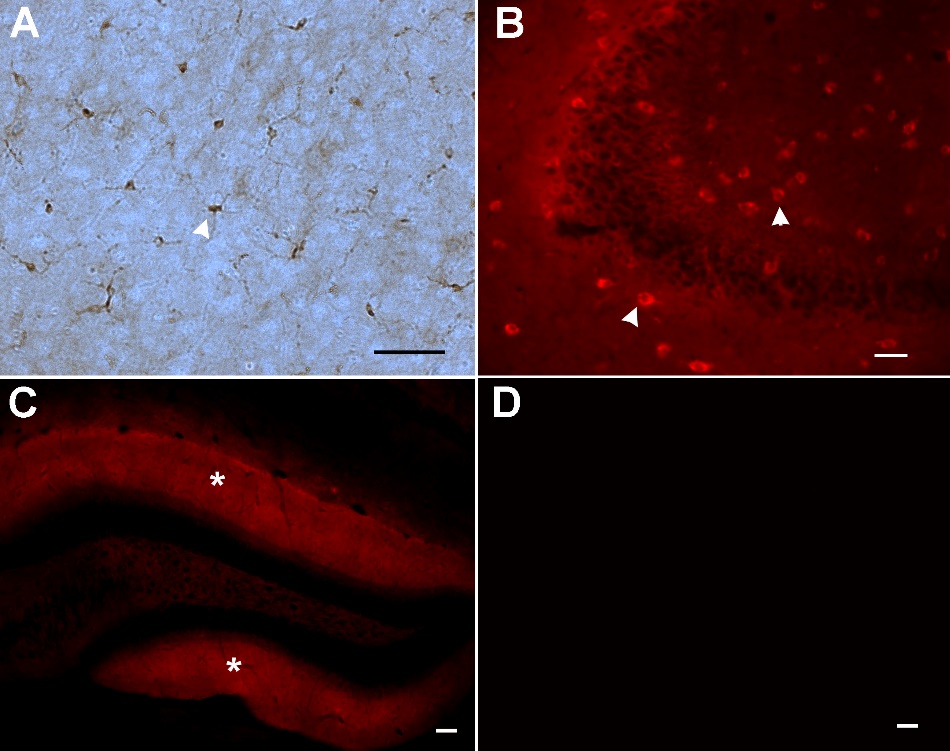


**Figure S1: C1q is detected in microglia, a subset of interneurons, and neuropil.**

(A) C1q immunostaining in microglia (arrowheads) of WT (16m) detected with HRP labeled anti rabbit antibody and DAB (brown). C1q immunofluorescence in (B) a subset of interneurons (arrowheads) in hippocampus in a WT 2m and (C) the molecular layer of dentate gyrus of WT (asterisks). No staining was observed in hippocampus from a C1q non-conditional KO (D) (4m). For B-D labeling was done using clone 27.1 antibody and Alexa555 fluorescent secondary antibody (red). Scale bar: 50um.


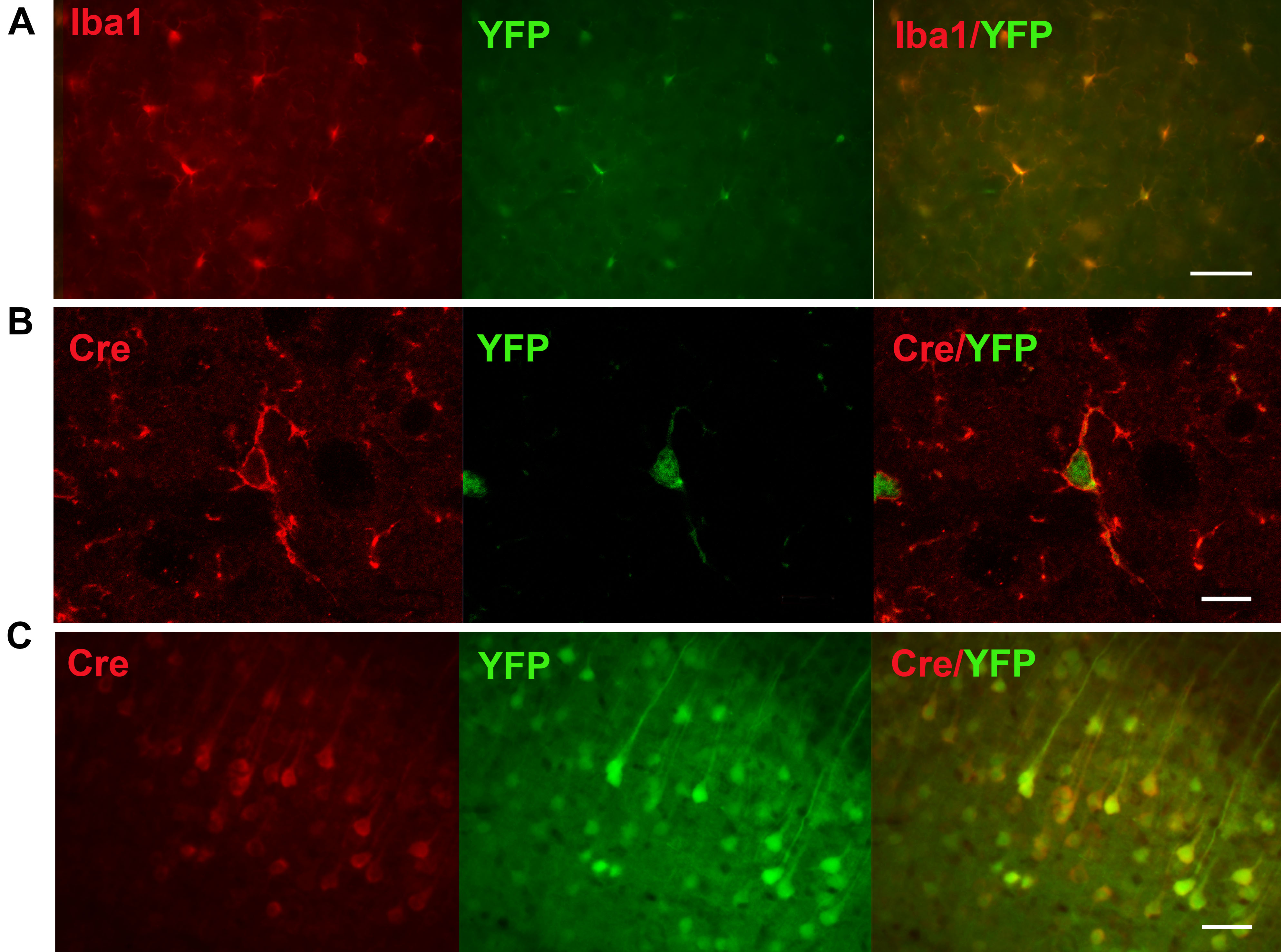


**Figure S2: Validation of cell specific expression in Cre deleter models.** A. Representative image of Iba1 (red, left), YFP (green, center) and merge (yellow, right) in *Cx3Cr1^CreERT2^* mice show all YFP labeled cells also have Iba1 reactivity and all Iba1 positive cells are YFP positive indicating that YFP is a marker for all microglia in our model. Scale bar: 50 um. B. Confocal image of anti Cre (red, left) and YFP (green, center) fluorescence in *Cx3Cr1^CreERT2^* mice(8m) show Cre and YFP colocalization in microglia of *Cx3Cr1Cre^ERT^* mice (merge, right). Scale bar: 10um. C. Anti Cre (red, left) colocalizes with YFP (green, center) in neurons of *Thy1^CreERT2^* mice. Merge (right) demonstrates expected complete colocalization of Cre and YFP. Scale bar: 50 um.

**
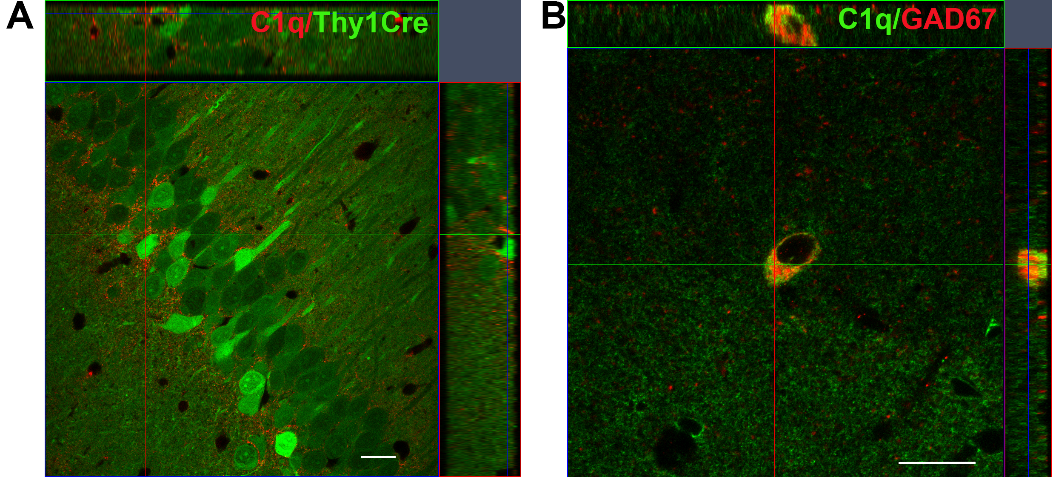
**

**Figure S3: C1q is juxtaposed to YFP-expressing Thy1+ neurons but is intracellularly colocalized with the interneuron marker, GAD67.** A. Orthogonal view of a representative confocal image of C1q staining (red) and YFP-Thy1^CreERT2^ (green) in *C1q ^FL^:Thy1^CreERT2^* mice (4m) shows lack of colocalization in Thy1 positive neurons. B. Orthogonal view of a representative image shows that C1q (green) colocalizes with GAD67 (red) in interneurons in wild type mice. Scale bars: 20um.

**
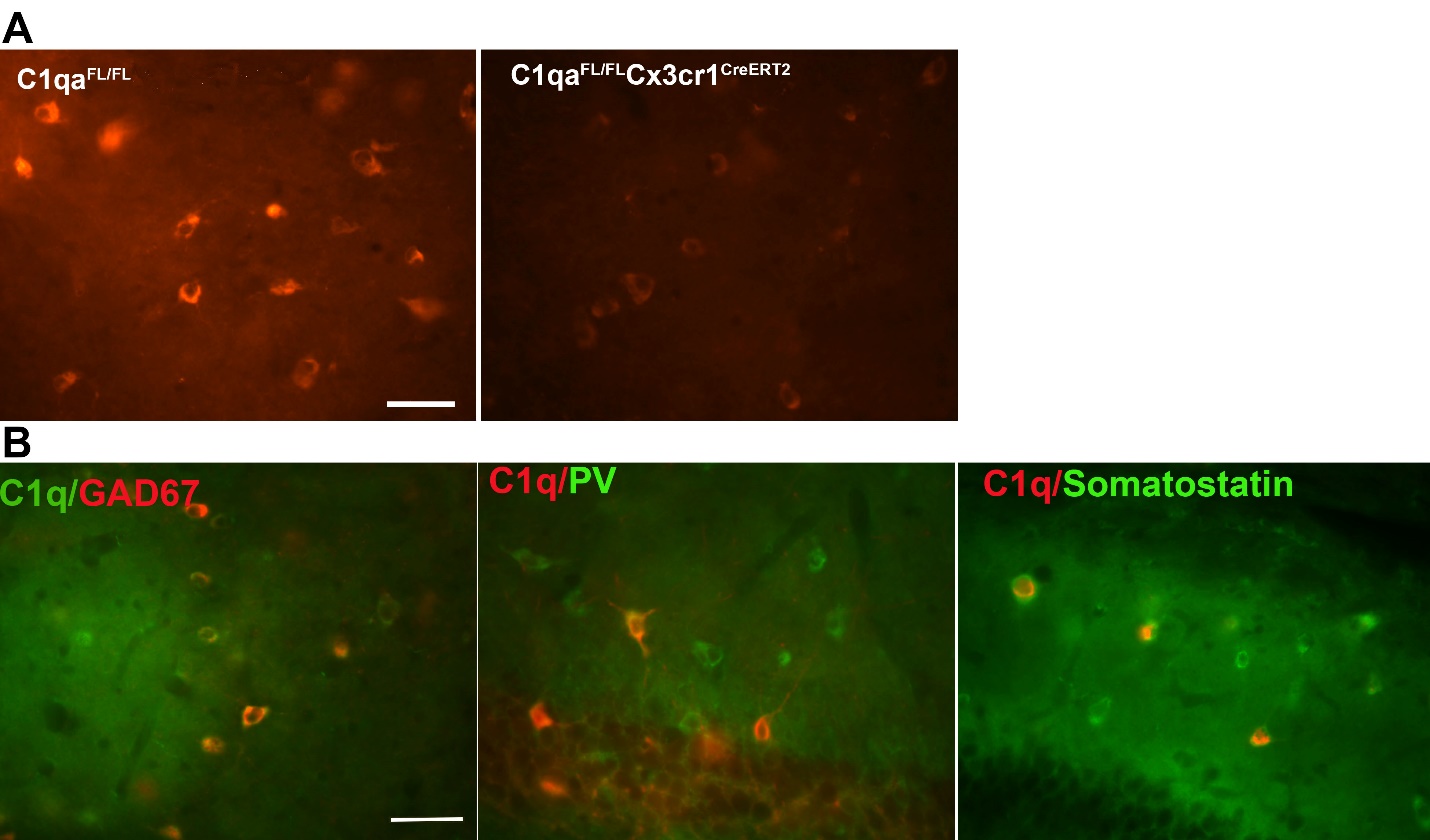
 Figure S4: C1q staining in interneurons.** A. C1q (red) is present in interneurons of *C1qa^FL/FL^:Cx3cr1^CreERT2^* (right panel) but is significantly decreased relative to *C1qa^FL/FL^* (left panel, littermate controls lacking *Cx3cr1^CreERT2^*) (1.5m). B. Colocalization of C1q (green) with GAD67, parvalbumin and somatostatin (red) in hippocampal interneurons in a 2m WT mouse. Scale bar: 50um.

**
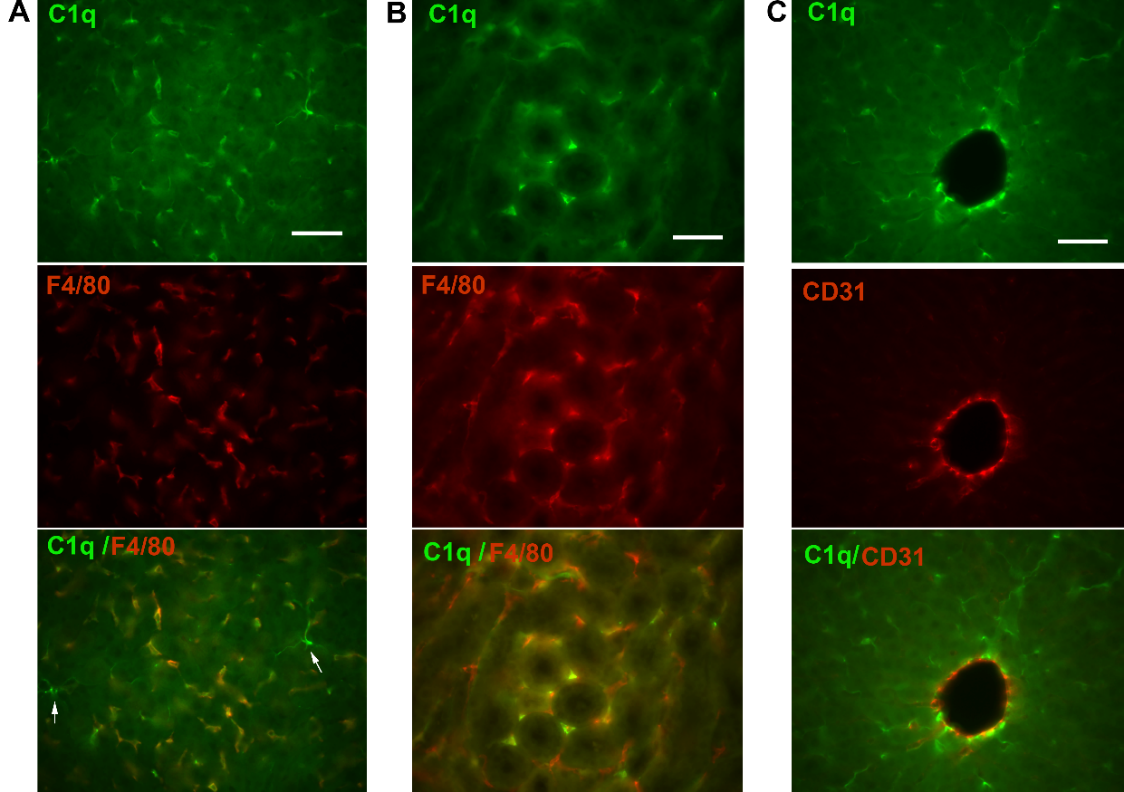
**

**Figure S5: C1q expression in liver and kidney of *C1qa^FL/FL^:Cx3cr1^CreERT2^* mice colocalizes with the macrophage marker F4/80, but not CD31.** Representative pictures of C1q (green) colocalization with F4/80 positive (red) macrophages in liver (A) and kidney (B) of a WT mouse (5m). C. Liver from a WT mouse (5m) stained for C1q (green) and endothelial cell marker, CD31 (red). Scale bar: 50um.


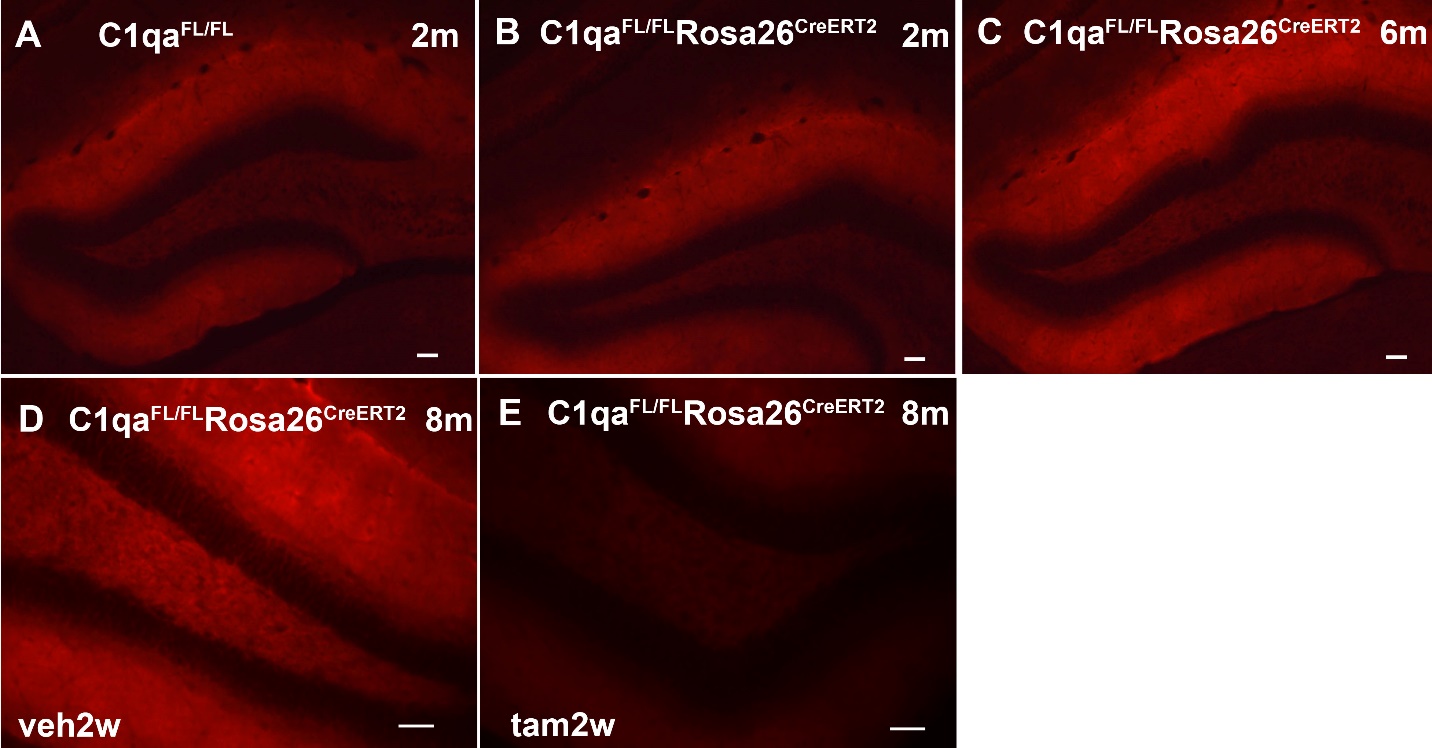


**Figure S6: No changes in C1q expression in *C1q^FL/FL^: Rosa26^CreERT2^* mice in the absence of tamoxifen but a decrease in C1q is induced by tamoxifen.**  C1q staining (red) in molecular layer of hippocampus in (A) untreated *C1q^FL/FL^* (littermate controls lacking Cre^ERT2^) or (B,C) *C1q^FL/FL^: Rosa26^CreERT2^* mice at 2 (A,B) and 6 months of age (C), or treated with vehicle (D) or tamoxifen (E) at 8 m (D,E) for 2 weeks. Scale bars: 50um.


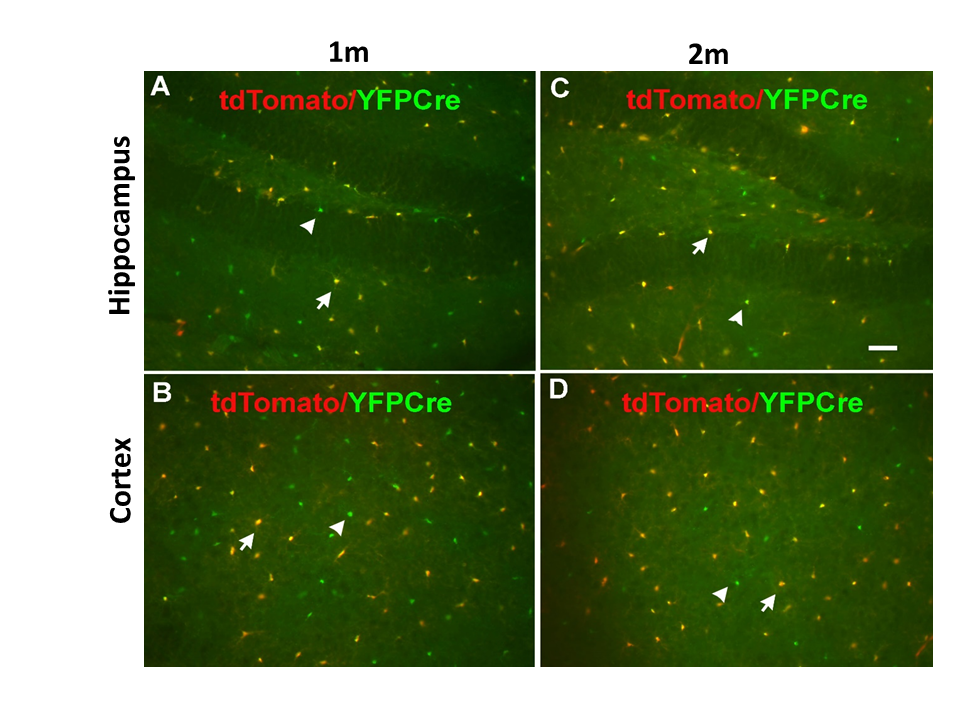


2m

**Figure S7:** ***Cx3cr1^CreERT2Wgan^* is “leaky” in the absence of tamoxifen.** Expression of tdTomato (which requires Cre activity) (red) in *Cx3cr1^CreERT2^* (microglia (YFP, green) of hippocampus (A,C) and cortex (B,D) of 1m (A,B) and 2m (C,D) *ROSA26-STOP-tdTomato:Cx3cr1^CreERT2^* mice in the absence of tamoxifen. Arrows and arrowheads show expression (yellow) or lack of expression of tdTomato (green), respectively. Scale bar: 50um. Representative pictures n=2 mice/age.
